# A systematic review protocol examining workplace interventions that aim to improve employee health and wellbeing in male-dominated industries

**DOI:** 10.1186/s13643-019-1260-9

**Published:** 2020-01-09

**Authors:** Paige M. Hulls, Rebecca C. Richmond, Richard M. Martin, Frank de Vocht

**Affiliations:** 1 Population Health Sciences, Bristol Medical School, University of Bristol, Bristol, UK; 2 MRC Integrative Epidemiology Unit, University of Bristol, Bristol, UK; 3 NIHR Bristol Biomedical Research Centre, University Hospitals Bristol NHS Foundation Trust and the University of Bristol, Bristol, UK; 4 NIHR School for Public Health Research, University of Bristol, Bristol, UK

**Keywords:** Male-dominated industries, Workplace interventions, Employee health and wellbeing, Occupational stress, Systematic review

## Abstract

**Background:**

The workplace environment potentially provides access to a large population who are employed, and it is an employer's responsibility to provide appropriate conditions for its employees. Whilst the aetiology of cardiovascular disease is multifactorial, it is generally acknowledged that working conditions, gender and age are involved in its development. Male-dominated industries (comprising > 70% male workers, e.g., agriculture, construction, manufacturing, mining, transport and technology) have a higher prevalence of health risk behaviours than other population subgroups. Working in a gender-dominated industry can impact an employee's health and wellbeing, particularly for the opposite sex. This systematic review examines workplace interventions that address the health and wellbeing of employees in male-dominated industries.

**Methods:**

We will include randomised controlled trials and studies with non-randomised intervention groups. The interventions must aim to improve employee physical and/or mental health and wellbeing implemented in the workplace in male-dominated industries. There will be no limits on date. The following electronic databases will be searched for published studies: Web of Science, Embed, MedLine, PsycInfo and the Cochrane Database. The search strategy will include free-text terms and MeSH vocabulary, including ‘male-dominated industries’, ‘workplace interventions’, ‘occupational stress’, ‘mental health’, ‘cardiovascular disease’, ‘blood pressure’, ‘body mass index’ and ‘exercise’. Two authors will independently select, review and extract data from studies that meet the inclusion criteria. The Cochrane's Risk of Bias tool will be used to assess risk of bias. We will perform structured summaries of the included studies and, if possible, conduct meta-analyses or construct an Albatross plot.

**Discussion:**

There are an increasing number of interventions designed to improve employee health and wellbeing in the workplace, but no prior review that systematically evaluates their effectiveness. A systematic review is required to prioritise the future implementation of those interventions found to be most effective.

**Systematic review registration:**

PROSPERO CRD42019161283

## Introduction

Workplace absence is estimated to be costing the UK economy £18 billion in lost productivity per year, a figure that is predicted will rise to £21 billion by 2020 and £26 billion by 2030 [1]. Stress is the most common cause of long-term absence and the second most common cause of short-term absence after minor illness [2]. Excessive volume of work and management style are two of the most common causes of workplace stress-related absence [3].

In a recent survey of 1021 UK organisations regarding 4.6 million employees, 83% of organisations reported taking action to address employee mental health in the workplace [3]. Common measures included phased return to work, employee assistance programmes (aims to help employees deal with personal issues that could adversely affect their work performance, health and wellbeing) and access to counselling. The World Health Organisation defined a healthy workplace as “*one in which workers and managers collaborate to use a continual improvement process to protect and promote the health, safety and well-being of all workers and the sustainability of the workplace*” [4]. Employers are considered to be in a suitable position to help support and promote employee health and wellbeing in several areas including nutrition, physical activity, disease management and workplace environmental changes [5–8].

Occupational factors—including high job strain, high effort-reward imbalance, low job control, low social support and overtime work—have been positively associated with cardiovascular disease (CVD) and its risk factors [9–15]. For example, employees working 55 h or more per week were reported to be at 30% increased risk of stroke versus those working standard hours [9].

Male-dominated industries have a higher prevalence of risky health behaviours [16, 17], including: smoking, overweight and obesity, high cholesterol, blood pressure and alcohol consumption [18]. Smoking and alcohol have been used to self-medicate stress-induced physiological effects [19] and job stress could increase smoking and drinking intensity [19–22]. However, the relationship between smoking status, alcohol consumption and occupational stress is mixed and inconclusive [19, 23, 24].

Male-dominated industries are commonly defined as comprising > 70% male workers and include agriculture, construction, manufacturing, mining, and transport and technology [25]. Similar industries are classified as male-dominated in most European countries [26], USA [27] and Australia [25, 28]. Working in a gender-dominated industry has been shown to impact an employee's health and wellbeing, particularly for the opposite sex [29]. Both males and females tended to have higher absence rates in workplaces numerically dominated by the opposite sex [30–32].

Although there is limited evidence, several interventions addressing employee health and wellbeing that have been trialled in male-dominated industries have indicated success. In white- and blue-collar workers in the construction industry with an elevated risk of CVD, there was a statistically significant beneficial effect on snack intake, fruit intake and smoking at six months in the intervention group [33]. Behaviour change is not only determined by personal factors, but also through the environment [34]. Authors concluded that the intervention could promote behaviour change among a population where risks of CVD and unhealthy lifestyles is likely to increase over the years. Focussing on mental health, a previous systematic review concluded that working conditions (including job strain and work–life balance) can have a significant impact on an employee's mental health as well as his/her job performance [35]. Providing training for senior and middle management, not only in improving access, but also in managing workload issues was crucial for intervention success. Moreover, there is very little evidence that considers the attitudes and experiences of employees and employers regarding health and wellbeing in male-dominated industries. Therefore, it would be beneficial to researchers, policy makers and companies within male-dominated industries to have access to a systematic review of the literature of the effectiveness of industry-specific interventions.

Existing systematic reviews focus on the health and wellbeing of employees. However, the extant published literature has not been necessarily based on workplace interventions or in male-dominated industries [25, 36–45]. To our knowledge, there is no published systematic review that examines the effectiveness of workplace interventions designed to address the health and wellbeing of employees in male-dominated industries.

The purpose of this project is to conduct a systematic review of the effectiveness of workplace interventions that aim to improve employee health and wellbeing in male-dominated industries, as described in this protocol paper.

## Objectives

The primary objectives of this review are to first, describe workplace interventions in male-dominated industries and evaluate the quality of the studies included; second, assess their effectiveness in improving employee health and wellbeing; and third, describe the acceptability of these interventions to employees and employers.

The secondary objectives of this review are to first, highlight similarities within the interventions and second, identify other male-dominated industries where the interventions could be successfully replicated.

## Methods and analysis

This protocol is registered in the PROSPERO database under number CRD42019161283 and is reported according to the Preferred Reporting Items for Systematic Review and Meta-Analysis Protocol (PRISMA-P, Additional file 1) [46].

### Review inclusion criteria

#### Types of employee health and wellbeing

Studies must include information and measures of physical and/or psychological health (health outcomes) and/or risk behaviours that may affect, or be the result of, physical and/or psychological health issues (health behaviours). Health behaviours are defined as “overt behavioural patterns, actions and habits that relate to health maintenance, health restoration and health improvement” [47]. There is no consensus on the definition of employee health and wellbeing and currently is a lack of commonly accepted and shared definitions. Interventions must include the three key dimensions of employee health; psychological, physical and social wellbeing as defined by Grant, et al. [48]. The first dimension is related to happiness and subjective positive experiences during work. The second dimension is related to physical wellbeing and health. The third dimension is related to the quality of relationships at work (both peer relations and hierarchical relationships).

#### Types of interventions

Interventions must aim to promote or improve employee health and wellbeing in male-dominated industries. The intervention must be implemented in the workplace and should either aim to alter the health behaviours of the employees, for example, alcohol consumption or smoking; make changes in their health and wellbeing, for example, self-reported health, occupational stress or absenteeism, or changes to the occupational environment, for example, provision of standing desks or changes to catering options. This distinction will be recorded in the data extraction form Additional file 2.

#### Types of studies

Studies must have quantitatively evaluated the workplace intervention using a controlled design, either a randomised controlled trial or studies with non-randomised intervention group allocation.

#### Types of participants

Participants are adults over the age of 18 years who are paid employees in an organisation within male-dominated industries, defined by the type of industry: construction, manufacturing, mining, transport, agriculture and technology (including information technology). There are no limits on study participants regarding gender, ethnicity, occupation or seniority.

#### Types of outcome measures

The primary outcomes of interest are intervention delivery; intervention uptake (engagement); intervention adherence; measures of physical health, including blood pressure, body mass index (BMI) and smoking cessation; and measures of psychological health or occupational stress using validated scales. Secondary outcomes of interest are: self-reported health behaviours; cognitive outcomes; social wellbeing; and knowledge, motivation and awareness of the measured health behaviours using validated scales and/or qualitative interviews.

##### Objective outcomes

- Adherence to intervention programmes; change in validated scales
- Adherence to intervention protocol
- Change in knowledge/skills regarding occupational stress
- Changes in pre-existing health conditions (physical and psychological health); changes in validated scales
- Access to healthcare professionals (physical, psychological health or occupational stress); percentage of appointments arranged; percentage of appointments missed
- Change in percentage of those taking regular medication

##### Self-reported outcomes

- Use/engagement of intervention; acceptability of the intervention
- Perception of intervention programme; perceived level of change in health/condition
- Change in validated scales for adherence to intervention programmes
- Social wellbeing; interactions and engagement with peers and/or colleagues
- Perception of health behaviours; self-efficacy to manage relevant changes
- Perceived ability to manage condition; self-efficacy to manage medication
- Perception of need to services and intention to attend services
- Application/use in information

### Search strategy

#### Electronic bibliographic databases

We will search the following databases: Web of Science, Embed, MedLine, PsycInfo and the Cochrane Database. The search strategy will include terms relating to ‘male-dominated industries’, ‘workplace interventions’, ‘intervention’, ‘office’, ‘occupational stress’, ‘burnout’, ‘mental health’, ‘depression’, ‘cardiovascular disease’, ‘blood pressure’, ‘hypertension’, ‘body mass index’, ‘diet’ and ‘exercise’. No limits for date of publication will be used, and publications in the English language will only be considered. This review will be of published peer review studies only and grey literature (including conferences, abstracts and dissertations) will not be considered in this systematic review. Whilst it has been suggested that grey literature should be considered in systematic reviews and meta-analyses, this review will only include peer-reviewed articles to collate the strongest evidence of effective interventions. Appendix shows the search strategy for MedLine. Prior to analysis, the search will be re-run to make sure all current studies are included in the analysis. Reference lists for all included studies will be searched for any additional interventions.

#### Study screening and selection

Titles and abstracts of studies selected via the search strategy and from other additional sources will be reviewed independently by two authors to identify studies that meet the inclusion criteria (see above). The full text will be retrieved and assessed for eligibility. Any discrepancies about the eligibility of any studies between the two authors will be discussed with a third reviewer and consensus reached. The reason for excluding papers will be recorded. The flow of papers through the selection process will be recorded using the PRISMA flow diagram.

#### Data extraction

A standardised, pre-determined form will be used to extract data from included studies. The following information will be extracted from the papers:

- Methods: study design, follow-up time points, type of workplace industry, study country and date of study
- Participants: total number, number by intervention arm, age (mean and range), percent male, trial inclusion and exclusion criteria and participant occupations
- Interventions: type of intervention (i.e., online or face–to-face) and content (i.e., duration, peer support or personalisation)
- Outcomes: primary and secondary outcomes as recorded above, and their follow-up time points
- Results: intervention effect sizes, mean differences, risk ratios, standard errors, *p*-values and confidence intervals
- Conflicts of interests declared by trial authors

### Analysis

#### Descriptive analysis

The included studies will have a narrative synthesis including the extracted baseline characteristics and the distribution of the outcomes. Study characteristics will be summarised in tabular form (study design, method, analysis, strengths and weaknesses) to allow authors to discuss the implemented approaches and comparable attributes.

It is expected that there will be limited opportunity to complete any meta-analysis, due to the range of different outcomes measured in the interventions and as well as the low number of interventions that fit our inclusion criteria. However, if studies have the same type of intervention and outcome measure, a meta-analysis will be completed using Stata v14.0 [49]. If there is moderate heterogeneity, then a fixed-effects meta-analysis will be used. If there is a high level of heterogeneity, then a random-effects meta-analysis will be more appropriate. Heterogeneity in intervention effects is caused by differences in study population, interventions received, follow-up length and other factors. Where formal meta-analysis is not possible due to differences in data collected, alternative analysis will be used based on minimal statistical information. Using total sample size and *p*-value, albatross plots will be generated to display the observed directions of effect and identify potential sources of heterogeneity [50]. The *p*-values will then be interpreted in relation to the study's sample size.

#### Assessing risk of bias

Studies will be evaluated using the Cochrane Collaboration's Risk of Bias Tool [51], which considers selection bias (random sequence allocation and allocation concealment), reporting bias (selective reporting), performance bias (blinding participants and personnel), detection bias (blinding of outcome assessment), attrition bias (incomplete outcome data) and other bias (other sources of bias). Studies will then be categorised into low risk of bias, unclear risk of bias or high risk of bias.

#### Measures of treatment effect

Summaries of intervention effects will be calculated for each study. Dichotomous data will be analysed as a risk ratio. Continuous data will be analysed using the mean difference of similarly scaled data. Ninety five percent confidence intervals will be used for all data.

#### Assessment of heterogeneity

Where formal meta-analysis is possible, studies included will be assessed for methodological and clinical heterogeneity using the I^2^ statistic. Given that the thresholds of interpreting I^2^ statistic can be misleading, Cochrane criteria on heterogeneity interpretation will be used [52]:

- 0% to 40%: might not be important
- 30%-60%: may represent moderate heterogeneity
- 50% to 90%: may represent substantial heterogeneity
- 75% to 100%: considerable heterogeneity

To further identify potential sources of heterogeneity, sensitivity analysis will be completed on study quality following the risk of bias tool, i.e., generalisation of randomisation sequence, allocation concealment and blinding. Publication bias will also be assessed using two methods; the Begg's rank correlation test for dichotomous outcomes and Egger's weighted regression method for continuous outcomes.

### Protocol amendments

In case of any changes to this protocol, the details of any changes will be outlined in the published final review and updated in PROSPERO. However, no further amendments to this protocol are foreseen.

## Discussion

There are an increasing number of health and wellbeing interventions being implemented in the workplace, but there are currently no systematic reviews that we are aware of that systematically appraise the evidence of effectiveness. This systematic review aims to identify successful strategies, components and/or interventions to help inform decision-making for future planning and implementation. It is expected that the systematic review will also inform the design and content of a workplace intervention that will be piloted in the UK construction industry. Findings from this systematic review will also be disseminated for peer-reviewed open access publications as well as at the relevant conferences. Limitations of this review include the inclusion of English language papers only; and the inclusion only of studies using a controlled design, either randomised controlled trials or intervention studies with non-randomised group allocation. A disadvantage of limiting to the English language only is that male-dominated industries from non-English speaking countries may be less represented in the review and any findings could only be applicable to particular countries. Including only controlled designs could exclude valuable information from workplaces within male-dominated industries where randomised controlled trials or non-randomised intervention group are not always possible. Such information includes the type of male-dominated industry, study environment and intervention content and/or target. Study-specific limitations will be discussed in detail in the published final review.

## Supplementary information

**Additional file 1.** PRISMA-P Checklist.

**Additional file 2.** Data extraction form.

## Data Availability

The datasets used and/or analysed during the current study are available from the corresponding author on reasonable request.

## Appendix

**Table 1:** MedLine sample search strategy

1	Male-dominated industry*.tw
2	Construction industry/
3	Manufacturing industry/
4	Exp coal mining/ or mining/
5	Agriculture/
6	Agriculture industr*.tw
7	Transport industr*.tw
8	Information technology/
9	Construction industr*.tw
10	Manufacturing industr*.tw
11	Mining industr*.tw
12	Information technology industr*.tw
13	IT industr*t.tw
14	1 or 2 or 3 or 4 or 5 or 6 or 7 or 8 or 9 or 10 or 11 or 12 or 13
15	Workplace/
16	Workplace intervention*.tw
17	Office*.tw
18	Intervention*.tw
19	Workplace program*.tw
20	15 or 16 or 17 or 18 or 19
21	Occupational stress/
22	Job related stress.tw
23	Work-related stress.tw
24	Occupational stress.tw
25	Burnout.tw
26	Mental health/
27	Anxiety disorders/
28	Depression/
29	Mental health.tw
30	Anxiety.tw
31	Performance anxiety.tw
32	Anxiety disorder.tw
33	Depression.tw
34	Anxiety/ or performance anxiety/
35	Burnout, professional/
36	21 or 22 or 23 or 24 or 25 or 26 or 27 or 28 or 29 or 30 or 31 or 32 or 33 or 34 or 35
37	14 and 20 and 36
38	Body mass index/ or body weight/
39	Body mass index.tw
40	Diet.tw
41	Exercise.tw
42	Fitness.tw
43	38 or 39 or 40 or 41 or 42
44	14 and 20 and 43
45	Cardiovascular diseases/
46	Cardio*.tw
47	Wellbeing.tw
48	Blood pressure/ or coronary disease/ or hypertension/
49	Blood pressure.tw
50	Hypertension.tw
51	Cholesterol.tw
52	45 or 46 or 47 or 48 or 49 or 50 or 51
53	14 and 20 and 52

## Abbreviations

BMI: Body mass index
CVD: Cardiovascular disease
